# Beyond ubiquitination: the atypical functions of Fbxo7 and other F-box proteins

**DOI:** 10.1098/rsob.130131

**Published:** 2013-10

**Authors:** David E. Nelson, Suzanne J. Randle, Heike Laman

**Affiliations:** Department of Pathology, University of Cambridge, Tennis Court Road, Cambridge CB2 1QP, UK

**Keywords:** Fbxo7/PARK15, F-box protein, ubiquitin, E3 ligase, Parkinson's disease, mitophagy

## Abstract

F-box proteins (FBPs) are substrate-recruiting subunits of Skp1-cullin1-FBP (SCF)-type E3 ubiquitin ligases. To date, 69 FBPs have been identified in humans, but ubiquitinated substrates have only been identified for a few, with the majority of FBPs remaining ‘orphans’. In recent years, a growing body of work has identified non-canonical, SCF-independent roles for about 12% of the human FBPs. These atypical FBPs affect processes as diverse as transcription, cell cycle regulation, mitochondrial dynamics and intracellular trafficking. Here, we provide a general review of FBPs, with a particular emphasis on these expanded functions. We review Fbxo7 as an exemplar of this special group as it has well-defined roles in both SCF and non-SCF complexes. We review its function as a cell cycle regulator, via its ability to stabilize p27 protein and Cdk6 complexes, and as a proteasome regulator, owing to its high affinity binding to PI31. We also highlight recent advances in our understanding of Fbxo7 function in Parkinson's disease, where it functions in the regulation of mitophagy with PINK1 and Parkin. We postulate that a few extraordinary FBPs act as platforms that seamlessly segue their canonical and non-canonical functions to integrate different cellular pathways and link their regulation.

## 2. Introduction

As with actors on a stage, the timely exit of cellular proteins is as important as their entrance; they must play their part at the appropriate moment and then depart on command. The ‘exit’ or destruction of proteins within the cell goes beyond the simple removal of proteins; it provides a means to achieve rapid activation, by the degradation of an inhibitor, or conversely, the inactivation of a given process faster than could be achieved by the synthesis of new inhibitor proteins. Furthermore, degradation is irreversible, imparting a unidirectionality that is absolutely fundamental to basic processes like the cell cycle, which requires the coordinated degradation of kinase inhibitors and activating cyclins, helping to ensure a single and complete round of replication of the genome [1,2].

The degradation of cellular proteins is not random but is directed by signalling pathways and carried out by the ubiquitin proteasome system (UPS) [3–5]. Proteins are marked for degradation by an initial post-translational modification or a series of modifications, such as phosphorylation, creating a ‘degron’ to which ubiquitin ligases are recruited to label targeted proteins with ubiquitin. These small, 8.5 kDa proteins, when attached as polymer chains, can direct proteins to proteasomes, large multi-subunit complexes with multiple proteolytic activities. Proteasomes essentially ‘recycle’ proteins by breaking them into short seven to eight amino acid polypeptides that are further broken down to their composite amino acids to be re-used or catabolized by the cell. The ubiquitination reaction itself is a well-defined process mediated by a cascade of ubiquitin-handling enzymes and has been reviewed in detail elsewhere [3,6–8]. The final step in this process is orchestrated by an E3 ubiquitin ligase. Its role is to recognize and bind degrons within a target protein and bring it into proximity with a ubiquitin-charged E2 protein, stimulating transfer of a ubiquitin moiety to recipient lysine residues within the target.

**Figure 1.**
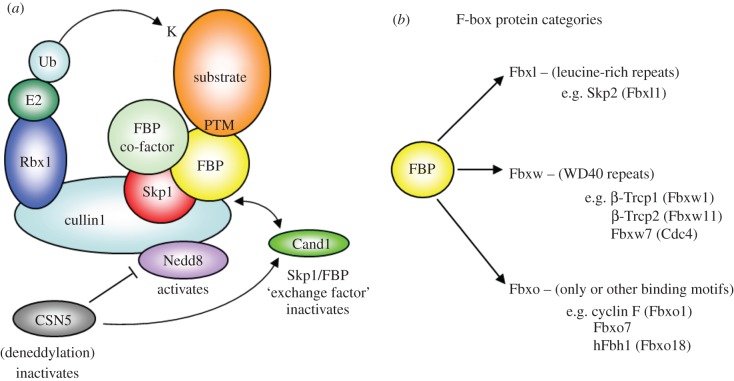
F-box proteins. (*a*) Schematic of active SCF complexes, which are neddylated (Nedd8 moiety attached to cullin1). FBPs can bind to the SCF holoenzyme alone, with accessory cofactors, or as a homo- or heterodimer allowing for dimeric SCF complex formation. The SCF complex is orientated so the lysine residue (K) in the substrate, usually recruited by FBPs after being PTM, is in close proximity to the ubiquitin moiety on the E2 enzyme. SCF complexes can be inactivated by deneddylation, at which point Cand1 can compete with Skp1/FBP for binding to cullin1, allowing for exchange of SCF subunits. (*b*) There are three classes of FBP, which are listed, along with examples from each group.

The array of targets regulated by E3 ubiquitin ligases is as broad as the proteome itself. One strategy the cell employs to handle the magnitude of this task is to express a large number of different E3 ligases. Indeed, the human genome encodes over 500 distinct E3s [9], roughly separated across two main families; the homologous to E6-associated protein (E6-AP) C-terminus (HECT) domain and the really interesting new gene (RING) finger domain E3s [3]. Another strategy that goes beyond merely increasing E3 numbers is to employ adaptor proteins that change the substrate specificity of the E3, which may enable a tailoring of substrate engagement with a ligase as per the changing needs of the cell. This strategy is typified by the Skp1-cullin1-F-box protein (SCF)-type E3 ubiquitin ligases, the largest group of multi-subunit E3 ligases within the RING finger domain family [5,10,11]. The F-box protein (FBP) family is fundamental to this flexible substrate-recognition, as they act as interchangeable docking sites for the ligase. The SCF holoenzyme is formed around a central cullin (Cul1) backbone, which provides a rigid scaffold, holding the E2 binding subunit, Rbx1, at a distance of approximately 50 Å from the substrate docking site (figure 1*a*) [8]. Substrate recruitment is the role of the FBP, and its tethering to cullin is mediated by Skp1.

In this review, we present a brief outline of the function and diversity of the FBP family, describing how they operate within the UPS system as conventional components of SCF-type E3 ubiquitin ligases. We will then discuss an array of alternative functions displayed by FBPs, as many are being reported to lead ‘double-lives’, operating outside the UPS system and independently of the SCF to regulate a diverse range of cellular processes. Lastly, we will focus on Fbxo7, an FBP implicated in cancer and neurodegenerative disease, whose atypical functions include regulating cell cycle, differentiation, proteasomal function and mitochondrial quality control. As illustrated by the exemplary case of Fbxo7, we propose that having both ubiquitination functions and non-canonical regulatory roles endows a subset of special FBPs with the potential to act as hubs to link the UPS to other cellular signalling networks.

## 3. F-box proteins

FBPs are defined by a 40–50 amino acid F-box domain that binds Skp1 [12]. After the discovery of the first FBP, cyclin F (Fbxo1) [13], further family members were identified using a yeast-two-hybrid screen for Skp1-interacting proteins and bioinformatics analysis for homologous sequences [14,15]. Without taking into account the various isoforms that may be produced, there are 69 human FBPs [16], and the number is significantly higher in other organisms, such as *Caenorhabditis elegans* and plants. FBPs are subdivided into three separate classes, Fbxw, Fbxl and Fbxo, based on their individual complement of protein interaction domains: WD40, leucine-rich repeats (LRR) and ‘other’, respectively (figure 1*b*) [17]. These three classes did not diverge from a single common ancestral gene, as might be expected. Instead the F-box phylogenetic tree is made up of two main groups with FBPs from all classes found in both, suggesting frequent swapping of protein interaction domains during their evolution [17]. The WD40 and LRR domains of Fbxw and Fbxl family members fold to create large surface areas for protein–protein interaction. WD40 repeats form a circularized propeller-like structure [18], while the successive repeats of LRR domains stack in a horseshoe formation [19]. The Fbxo class, on the other hand, features a broad array of different interaction domains, including in between-ring domains (IBR), TRAF-domain like motifs and proline-rich regions (PRR), among others. Although these domains from all classes of FBPs directly recruit substrates, in some cases an additional cofactor is essential or can change the substrate specificity of the FBP. For example, Fbxl1 (Skp2) requires Cks1 to bind the Cdk inhibitor p27 or the pRb-related protein, p130 for ubiquitination [20,21]. However, Cks1 is not needed for the recruitment of other Skp2 substrates.

Quite how the most appropriate FBPs are ‘selected’ and loaded onto a cullin scaffold is not fully understood, but it is thought to be regulated by two processes: availability and exchange. The pool of available FBPs is likely to be controlled by a combination of transcriptional regulation, targeted stabilization/degradation and auto-ubiquitination in the absence of their substrates [22,23]. For example, the muscle catabolism regulator, Atrogin-1 (Fbxo32) is controlled transcriptionally. Its expression is enhanced under starvation conditions, promoting muscle atrophy, but is suppressed by testosterone, stimulating increased muscle mass [24]. On the other hand, the putative tumour suppressor, Fbxo31, is regulated post-translationally. It is stabilized in response to DNA damage and targets ATM-phosphorylated cyclin D1 for UPS-dependent degradation, thus bringing about a G1 cell cycle arrest [25]. Auto-ubiquitination in the absence of a suitable substrate is a process thought to be very common among FBPs. It provides a means by which ‘idle’ FBPs target themselves for UPS-dependent degradation. This is typified by β-Trcp2 (Fbxw11), a regulator of the Wnt and NF-κB signalling pathways, which is intrinsically unstable under basal conditions but has been shown to be stabilized by the induction of one of its substrates, the phosphorylated form of inhibitor of NF-κB alpha (IκBα) [26]. The variety of these mechanisms provides the means to upregulate required FBPs in response to intrinsic and extrinsic signals, while continually clearing those that are no longer required, ensuring that the available pool addresses the needs of the cell at any given time. The mechanism by which one FBP is ‘switched-out’ and replaced by another has recently been reported and is controlled by the ‘exchange factor’ Cand1 [27,28]. By increasing the dissociation rate of Skp1 : FBP complexes from the cullin scaffold, without affecting the kinetics of its reassembly, Cand1 keeps the spectrum of SCF activity within the cell dynamic and capable of responding to a changing cellular environment.

**Figure 2.**
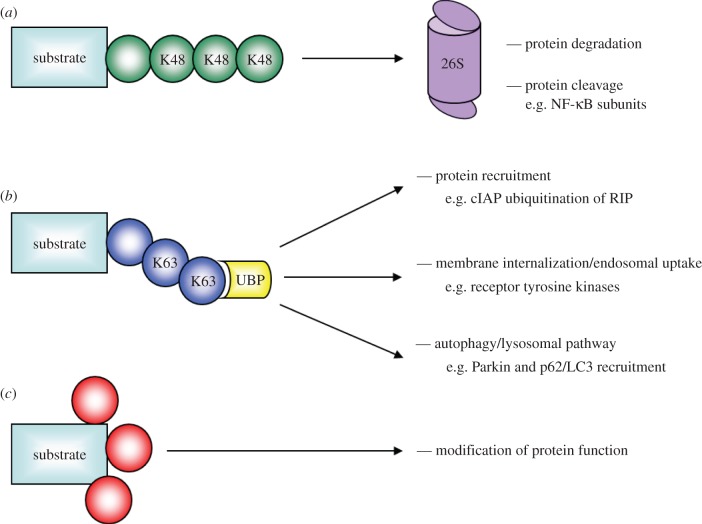
Common ubiquitin linkages and their consequences. (*a*) Polyubiquitin chains linked via lysine 48 (K48) to the preceding ubiquitin molecule usually target proteins for degradation via the 26S proteasome. Poly-K48 chains can also result in proteasome-mediated protein cleavage such as in the case of the NF-κB subunits, p100 and p105, being processed into p52 and p50, respectively. (*b*) Polyubiquitination via lysine 63 (K63) is also common. This type of ubiquitination usually acts as a scaffold for UBPs that recruit other protein complexes, and also mediates alternate outcomes such as changes to cell signalling (e.g. ubiquitinated RIP can recruit NF-κB signalling pathway components), activation of the endosomal pathway (e.g. resulting in internalization of membrane proteins such as receptor tyrosine kinases) and activation of the autophagic/lysosomal pathway (e.g. resulting in degradation of target proteins and organelles independently of the proteasome). (*c*) Monoubiquitination, or multi-monoubiquitination, can result in changes to substrate function, localization or protein binding.

FBPs interact with and ubiquitinate their own particular panel of substrates, usually showing a preference for post-translationally modified (PTM), often phosphorylated, proteins. In this way, signal transduction networks that use protein kinases (e.g. GSK3β, Cdks, IKK) can engage a UPS response. Recognition by an FBP can be via a single PTM or cumulative PTMs of the substrate on multiple sites. This latter scenario, which creates a switch that senses and responds to a threshold of modifications, is used by the yeast Cdk inhibitor, Sic1. Phosphorylation on any six of its nine phospho-acceptor sites stimulates its ubiquitination by SCF^Cdc4^ as part of the transition from G1 to S phase [29]. Thus as part of an SCF, FBPs ‘translate’ a PTM from upstream signalling pathways into a ubiquitination response, and that signal can be further acted upon and/or stratified. Ubiquitin can be conjugated as individual moieties to single or multiple sites within a protein (mono and multi-monoubiquitination, respectively) or attached as a polyubiquitin chain (figure 2). Such chains can differ in both length and topology, which forms the basis of the ‘ubiquitin code’ (reviewed in [30]). Ubiquitin contains eight residues (Met1, Lys6, Lys11, Lys27, Lys29, Lys33, Lys48 and Lys63) that are capable of forming an isopeptide bond with the C-terminal glycine residue (Gly76) of the preceding ubiquitin molecule in a nascent chain. Chains can be constructed using either a single type of linkage (homogeneous), several types (mixed) or be branched. The ‘deciphering’ of this code is due to the fact that different ubiquitin chain topologies recruit distinct ubiquitin binding proteins (UBPs) with activities that bring about changes to protein activity, location and/or levels [30]. The two best understood types of polyubiquitin chain linkage are those assembled on Lys48 or Lys63. Lys48 polyubiquitination is the most common linkage used in mammalian cells and is almost exclusively associated with the targeting of proteins for proteasomal degradation. It is the only essential lysine residue in yeast ubiquitin [31]. By contrast, Lys63 chains are generally not associated with UPS-based proteolysis but do play a major role in regulating the destruction of proteins and organelles by the lysosomal/autophagy pathway [32]. They are also used in other signalling networks, including the DNA damage response and the NF-κB signal transduction pathway [33,34]. In these instances, they are used to build scaffolds or ‘platforms’ for protein recruitment via UBPs.

The ability of an E3 enzyme to conjugate one type of polyubiquitin chain or another on its substrate is largely dictated by the spectrum of E2 enzymes with which it interacts [30,35,36]. With few exceptions, this appears to limit SCF-type E3 complexes to participating almost exclusively in the production of Lys48 polyubiquitin chains, thus promoting degradation. However, in some cases F-box-dependent Lys48 modification does not lead to complete proteolysis. SCF^β-Trcp^-induced poly-Lys48 ubiquitination can stimulate the 26S proteasome-dependent processing of the NF-κB transcription factors p100 and p105 to p52 and p50, respectively [37–39]. Interestingly, a growing number of reports have shown that FBPs are capable of participating in a broader range of ubiquitin–conjugation reactions. For example, Chen *et al.* [40] have shown that monoubiquitination of CTP : phosphocholine cytidylyltransferase (CCTα) by Fbxl2 targets it for endosome-lysosomal degradation. Another study has also suggested that β-Trcp has the capacity to direct Lys63 as well as Lys48 polyubiquitination of the interferon α/β receptor 1, when paired with an appropriate E2 enzyme *in vitro* [41].

In sum, through recognition of PTMs in its substrates, FBPs can use the SCF machinery to codify a ubiquitin-based response thus relaying and diversifying a signal into downstream cellular pathways.

## 4. SCF-independent functions of F-box proteins

The study of FBPs to date has focused on their SCF-dependent functions, even though the majority of FBPs in humans and other species remain ‘orphans’. The identification of substrates for these orphans remains a major endeavour for the field. However, an additional, perhaps under-appreciated consideration is that the many FBPs within a cell must compete for binding to the cullin scaffold and consequently may not be able to participate readily in ubiquitination reactions. It is therefore conceivable that these unengaged subunits may be free to participate in other reactions. In yeast, several FBPs have been found bound to Skp1, but not as part of an SCF complex. Instead FBP–Skp1 dimers participate in processes like centromere complex assembly and the recycling of endosome components (reviewed in [42]). Although the first descriptions of SCF-independent functions were in yeast, such roles for mammalian FBPs have also been reported [43]. For example, Emi1 (Fbxo5) functions as an SCF-independent suppressor of APC/C activity. Emi1 negatively regulates APC/C activity by binding to its activators, Cdc20 and Cdh1, which recruit APC/C substrates [44,45]. Thus, Emi1 prevents DNA re-replication and helps to link DNA replication to mitosis (reviewed in [44]).

The presence of an F-box domain itself is no guarantee that a protein will function as part of an SCF. Fbxo38 (MoKA) uses its F-box domain to interact with Kruppel-like transcription factor 7 (Klf7) [46]. Klf7 plays a key role in the development of the mammalian central nervous system by regulating differentiation, and maintaining cell cycle arrest, of post-mitotic neuro-progenitor cells [47]. Fbxo38 supports Klf7 in this role by acting as a transcriptional cofactor at the promoter of the cell cycle inhibitor p21^WAF1/Cip1^ [46]. Although it can bind to Skp1, to date an SCF-dependent role for Fbxo38 has yet to be identified. Moreover, this study raises the possibility that other FBPs might also use their F-box domains in transcriptional regulation.

Alternative functions for FBPs are not limited to simple binding interactions with other proteins, as they may also possess distinct and intrinsic enzymatic activities. hFbh1 (Fbxo18) has been shown to operate as a DNA helicase and is important for the maintenance of genomic stability through regulating homologous recombination [48,49]. The purified hFbh1 protein has DNA helicase activity, and strikingly this capacity is maintained when it is part of an SCF ligase [50]. However, as hFbh1 is an orphan FBP, the biological significance of linking ubiquitination activity to a helicase remains to be determined [49].

These examples illustrate that several FBPs, to date about 12% and usually of the Fbxo class, have activities beyond ubiquitination (table 1). We speculate that this class of special FBPs provides a means to link modification with ubiquitin to other enzymatic or functional interactions. Below, we will expand on Fbxo7 as a case in point for such FBPs, as it has well-defined SCF-dependent and independent activities; and furthermore, it is important in human health, having been linked to two diseases, cancer and Parkinson's disease (PD), and to alterations in red blood cell parameters.

**Table 1.** FBPs with SCF-independent functions (abbreviations are *Saccharomyces cerevisiae*, *Caenorhabditis elegans*, *Schizosaccharomyces pombe* and *Fusarium oxysporum*).

FBP	organism	function	references	additional SCF function
cell cycle regulation				
Fbxo7	mammals	cell cycle, proteasome and mitophagy regulator	[51,52]	yes
Emi1 (Fbxo5)	mammals	suppressor of APC/C activity	[44,45,53]	possible
Emi2 (Fbxo43)	mammals	suppressor of APC/C activity	[54]	unknown
Cyclin F (Fbxo1)	mammals	promotes nuclear localization of cyclin B1	[55]	yes
Ctf13p	*S. cerevisiae*	structural component of the CBF3 kinetochore complex	[56]	no
transcription/translation				
MoKA (Fbxo38)	mammals	transcriptional cofactor for KLF7	[46,47]	unknown
hFbh1 (Fbxo18)	mammals	DNA helicase	[48–50]	yes
KDM2B (Fbxl10)	mammals	histone demethylase	[57]	unknown
KDM2A (Fbxl11)	mammals	histone demethylase, inhibitor of NF-κB	[58]	unknown
Elongin A	mammals	translation elongation activator	[59,60]	no, but E3 activity via Cul5
FOG-2	*C. elegans*	translational repressor, binds GLD-1	[61]	no
intracellular trafficking				
Roy1/Ymr258c	*S. cerevisiae*	inhibits Ypt52 and consequently intracellular trafficking	[62]	no
Rcy1	*S. cerevisiae*	v-SNARE recycling	[63]	no
Pof6	*S. pombe*	endocytosis, cytokinesis and cell division (Rcy1 homolog)	[64]	yes
other				
Pof14	*S. pombe*	inhibits Erg9, a squalene synthase involved in ergosterol synthesis	[65]	yes
Mfb1	*S. cerevisiae*	mitochondrial morphogenesis - promotes fission	[66]	unknown
Mdm30	*S. cerevisiae*	mitochondrial morphogenesis - prevents fission	[67]	yes
Frp1	*F. oxysporum*	FBP required for pathogenicity in tomato wilt disease	[42,68,69]	yes

## 5. Fbxo7: gene and protein structure

**Figure 3.**
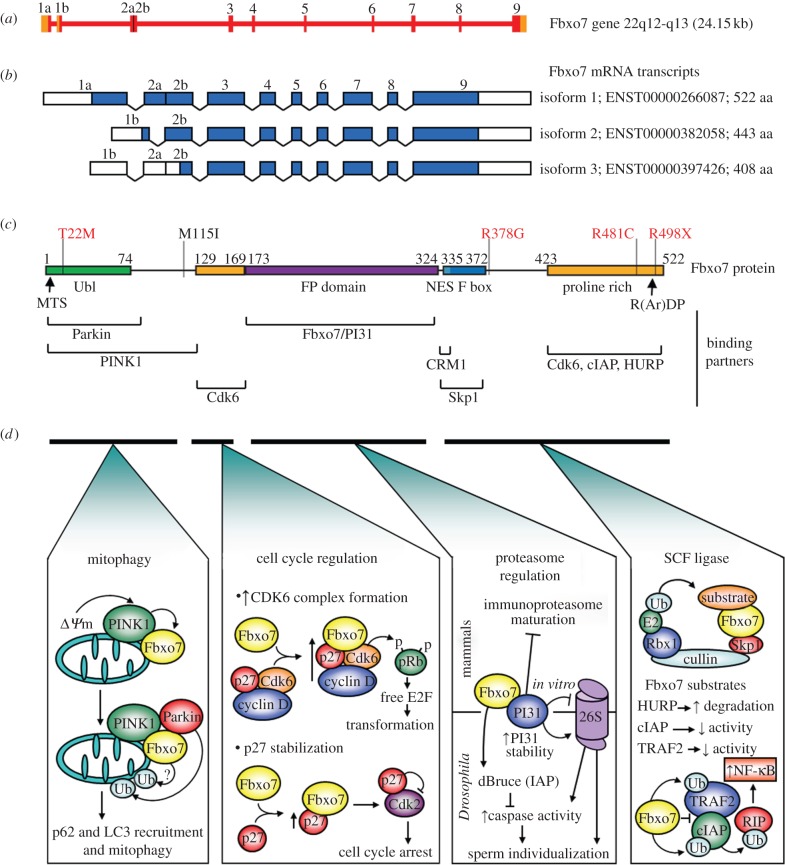
Human Fbxo7 gene and protein structure. (*a*) *FBXO7* gene organization. Orange and red boxes are untranslated and translated portions of exons, respectively, with exon numbers, as indicated. (*b*) Protein coding Fbxo7 mRNA transcripts, with Ensembl accession numbers and protein size. (*c*) Fbxo7 isoform 1 protein structure. Protein binding partners are listed below the domains with which they interact. Pathogenic mutations associated with PD are in red, and SNPs associated with red blood cell parameters in black. MTS, mitochondrial targeting signal; Ubl, ubiquitin-like domain; FP, Fbxo7/PI31 interacting domain; R(Ar)DP motif, where Ar is an aromatic amino acid. (*d*) Fbxo7 functions described for each domain relative to the protein schematic above. They are subdivided into canonical (SCF ligase) and non-canonical functions (mitophagy, cell cycle regulation and proteasome regulation). See text for details.

Fbxo7 was first identified as an FBP in 1999 [14,15], and the first report on its function was its canonical ubiquitination of HURP in 2004 [70]. However, a facilitating role for Fbxo7 in promoting cell cycle progression (discussed in §7) was discovered a year later and was the first clue that it had expanded activities [51]. This study also reported the basic domain structure of isoform 1 of Fbxo7 (figure 3*c*). In addition to the signature F-box domain, it contains a ubiquitin-like (Ubl) domain at its N-terminus and an unstructured PRR, used to recruit substrates, at its C-terminus [51]. While the F-box domain of Fbxo7 is most closely homologous to that of fellow FBPs, Fbxo9 and Fbxo11, its closest relative, proteasome inhibitor 31 (PI31), is in fact, not an FBP at all [71]. PI31 is a regulator of immunoproteasome maturation and an inhibitor of 20S proteasomes *in vitro* [72,73], and shares a common domain organization with Fbxo7 having a C-terminal PRR, containing a conserved R(Ar)DP motif, which is present in all orthologues of the two proteins [71]. Perhaps more significantly, both proteins share a core globular domain, designated the Fbxo7/PI31 (FP) domain that provides two distinct interaction surfaces that can mediate their homo- or heterodimerization [71]. Their relationship will be discussed in §8, but their common organization and shared domain structure raise the intriguing possibility that Fbxo7 was first a proteasome regulator that later gained the ability to ubiquitinate proteins.

The *FBXO7* gene is located on chromosome 22q12-q13 in humans and is composed of nine exons spanning a region of approximately 24.15 kb (figure 3*a*). From this, 10 transcripts have been identified and annotated by Ensembl [74], as well as a further two non-coding transcripts, with differing nomenclature, suggested by Di Fonzo *et al.* [75]. The Ensembl annotated transcripts include three complete protein coding isoforms (figure 3*b*), and an additional seven transcripts, which are incomplete, non-coding or presumed to undergo nonsense-mediated decay. The three coding transcripts comprise of isoform 1, which is the most abundantly expressed form of the protein in most tissues and cultured cell lines, and two shorter transcripts, isoforms 2 and 3. Isoform 2 contains exons 3–9 of isoform 1, but has an alternative 5′ exon. This alternative exon, known as 1b, splices directly to exon 2b, skipping exon 2a, and produces a 443 amino acid protein with an entirely different N-terminal end, lacking the Ubl domain seen in isoform 1. This version of the protein is also detectable in mammalian tissues and cell lines. Although less well studied than isoform 1, isoform 2 interacts with some of the known Fbxo7 substrates [51,76,77]. Isoform 3 also contains exons 1b, 2a and 2b; however, protein translation starts from an alternate methionine distinct from that of isoform 2. Intriguingly, the initiating methionine of isoform 3 is located at amino acid 115 (according to isoform 1 numbering) and its coding is affected by a common single nucleotide polymorphism (SNP; rs11107; M115I) that changes the nucleotide sequence from ATG (methionine) to ATA (Isoleucine). This SNP, along with several others in *FBXO7*, is associated with changes to red blood cell volume in humans [78–80]. Although no evidence as yet has been published showing protein expression of isoform 3, this would suggest that people homozygous for the ATA allele would completely lack expression of this isoform.

## 6. Fbxo7 as an E3 ubiquitin ligase

Currently, the capacity of Fbxo7 to function as a canonical FBP has been described for three substrates: HURP, cIAP1 and TRAF2 (figure 3*d*) [70,76,81]. HURP is a cell cycle-regulated protein associated with the mitotic spindle, where it regulates chromosome congression [82,83]. HURP ubiquitination by SCF^Fbxo7^ is preceded by its multisite phosphorylation by cyclin B/Cdk1 [70]. HURP was originally identified as a putative oncogene in hepatocellular carcinoma (HCC) and mechanistically has been shown to be a negative regulator of the tumour suppressor, p53 [84]. This raises the possibility that Fbxo7 could function as a tumour suppressor in HCC by negatively regulating HURP thus bolstering p53 activity, although this has not yet been investigated.

cIAP1 is a member of the IAP family, and it contains a C-terminal RING domain, enabling it to function as an E3 ubiquitin ligase [85]. It is this property of cIAP1 that allows it to inhibit apoptosis, both by targeting pro-apoptotic proteins, such as SMAC/DIABLO, for proteasomal degradation, and by stimulating anti-apoptotic NF-κB activity [85,86]. cIAP1 contributes to NF-κB signalling by forming part of the tumour necrosis factor-receptor signalling complex (TNF-RSC). Here, it interacts with TRAF2, another ubiquitin ligase and together they polyubiquitinate receptor interacting protein 1 (RIP) with Lys63-chains [86,87], producing a platform for the recruitment and activation of the inhibitor kappa B kinase (IKK) signalosome. IKK phosphorylates IκBα, stimulating its degradation and the concomitant release of NF-κB transcription factors into the nucleus. Using an siRNA screen targeting ubiquitin conjugating and de-conjugating enzymes, Kuiken *et al.* [81] recently identified Fbxo7 as an inhibitor of NF-κB activity, an effect that was mediated by the ubiquitination of cIAP1 and TRAF2. Thus, Fbxo7 has the potential to sever the link between the TNF-RSC core complex and the IKK signalosome, attenuating NF-κB activation. Dysregulation of NF-κB signalling has long been associated with cancer and oncogenesis and more recently has been linked to neurodegeneration and PD (reviewed in [88]). While it is tempting to speculate that the relationship between Fbxo7 and NF-κB signalling may be instrumental to its role in various pathologies, none of the currently known PD-associated Fbxo7 mutants (see §9) appear to affect NF-κB-dependent transcription *ex vivo* [81], and the ability of Fbxo7 to affect NF-κB signalling in a cancer context has yet to be probed.

A recent quantitative analysis of the Cul1 proteome revealed that SCF^Fbxo7^ was the fifth most abundant SCF ligase in cultured cells [89], placing it in the ranks of FBPs like Skp2, which has at least 26 reported substrates [90], and β-Trcp1 (Fbxw1A), which has 10 verified substrates [91]. As evidenced by these three substrates, Fbxo7 has the potential to impact upon disease-associated signalling pathways, and given the association of Fbxo7 with human diseases, a more complete reckoning of Fbxo7 ubiquitination substrates is eagerly awaited. However, to truly comprehend Fbxo7 function and its role in disease, a full understanding of its alternative activities is also warranted.

## 7. Fbxo7 as a regulator of the cell cycle

The first SCF-independent function for Fbxo7 was uncovered when the protein was identified as interacting with an oncogenic viral cyclin [51]. Viral cyclins bypass normal cell cycle regulators and robustly activate G1 Cdks, promoting S phase entry [92]. Fbxo7 was found to associate specifically and directly via a bipartite interaction with Cdk6 (figure 3*c*). In this capacity, it functions as an assembly scaffold for the formation of cyclin D/Cdk6 complexes, rather than causing the ubiquitination of either subunit (figure 3*d*) [51]. As these G1/S regulators are themselves proto-oncogenes, as a direct positive regulator, it was thought that Fbxo7 could also be a putative oncogene. This was borne out in experiments showing that over-expression of Fbxo7 in mouse fibroblasts triggered changes associated with cellular transformation, including tumour formation in nude mice. In addition, Fbxo7 over-expression was observed in human tumour biopsies from lung squamous cell carcinoma and colon adenocarcinoma but was virtually absent from corresponding normal tissue, suggesting that Fbxo7 may be oncogenic in these tissue types [51].

In a stringent test of its effects on proliferation, differentiation and transformation in primary cells, exogenous Fbxo7 expression was introduced into murine haematopoietic stem and progenitor cells (HSPCs) [93]. Haematopoietic cells were tested because of the selectivity of Fbxo7 for Cdk6, and because of the critical roles for Cdk6 in haematopoiesis: Cdk6 KO mice have thymic and splenic hypoplasia and reduced numbers of erythroid cells [94,95]. In this setting, increased Fbxo7 expression reduced both colony formation and proliferation of WT HSPCs along the granulocyte/macrophage lineages. However, in p53 null HSPCs, Fbxo7 expression enhanced proliferation in a growth factor-dependent manner and was also able to induce the formation of T-cell lymphomas when these cells were used to reconstitute irradiated mice [93]. Although this study did not test for Cdk6 dependence of the transformation, it nonetheless suggested that Fbxo7 has oncogenic capacity, which is held in check by p53.

Surprisingly, Fbxo7 has also been found to bind directly and to stabilize the levels of a second cell cycle regulator that acts at the G1/S transition, the Cdk inhibitor, p27. Cip/Kip inhibitors (p21, p27 and p57) usually function as inhibitors of Cdks, but they can operate as assembly and nuclear import factors for Cdk4 and 6. These competing functions of p27, inhibitor versus assembly factor, were originally postulated to be dependent on the stoichiometry of the Cip/Kip proteins relative to cyclin D/Cdk4/6 complexes [96]. However, more recent data would suggest that tyrosine phosphorylation of p27 converts it from a ‘bound inhibitor’ into a ‘bound non-inhibitory’ assembly factor [97,98]. The ability of Fbxo7 to act as a scaffold for the assembly of cyclin D/Cdk6/p27 complexes raises the possibility that it may facilitate p27 phosphorylation. It was an interaction with p27 that was thought to prevail and ultimately drive the phenotypic effects of Fbxo7 expression in a different haematopoietic cell type. In a separate study, it was shown that reducing Fbxo7 expression increased the proliferation rate in B cells by shortening the duration of the G1 phase of the cell cycle [99]. In this cell type, decreased Fbxo7 appeared to have no effect on the assembly or activity of cyclin D/Cdk6 complexes. Instead, a reduction in p27 levels was observed, along with enhanced Cdk2 activity. This study also demonstrated the ability of Fbxo7 to influence differentiation as well as cell cycle, two process that are thought to be closely coupled, but separable in lymphocytes [100–104]. Expressing Fbxo7 in Ba/F3 cells caused an apparent ‘de-differentiation’, as evidenced by a reversal in the expression of cell surface antigens from a more mature expression state to a less mature one. This effect was separable from the ability of Fbxo7 to regulate the cell cycle as over-expression of p27 alone, while altering cell cycle length, did not affect surface marker expression [99].

Together these studies illustrate how the activities of Fbxo7 are highly context-dependent: Fbxo7 can promote cell cycle entry by driving Cdk6 assembly or inhibit cell cycle progression by stabilizing p27. We speculate that this will be a consequence of the cell cycle regulatory circuitry in individual cell types or its stage of differentiation. As the outcome of Fbxo7 activity cannot be easily predicted by the mere abundance of its targets, we suggest that additional parameters, perhaps in the form of post-translational modifications of p27, Cdk6, Fbxo7 itself or other unknown factors, dictate the outcome of its participation at the G1/S boundary.

## 8. Fbxo7 as a regulator of proteasome activity

As with all FBPs, the link between Fbxo7 and the UPS could theoretically be limited to its ability to ubiquitinate proteins destined for proteasomal degradation. However, the presence of a Ubl domain in isoform 1 of Fbxo7, a motif commonly found in regulators of the proteasome, and its dimerization with PI31, a known proteasome regulator, suggest that Fbxo7 may itself act as a regulator of the proteasome (figure 3*d*). Nothing has been reported yet on the Ubl of Fbxo7 with regard to proteasome regulation; however, Fbxo7 binds with high affinity to PI31 [71], which was first characterized biochemically as a simple proteasome inhibitor [105]. PI31 binds the α-subunits of the 20S barrel via a conserved C-terminal HbYX motif, blocking access of substrates to the catalytic channel *in vitro* [72,106]. However, in intact cells, PI31 apparently does not inhibit proteasome activity but rather regulates maturation of the immunoproteasome, an inducible version of the proteasome with altered and enhanced proteolytic activity [73]. To date, the connection, if any, between PI31 and Fbxo7 in regulating mammalian proteasomal activity has not been published. This may be because in cultured cells, the localization of the two proteins is largely distinct. PI31 has been shown to localize almost entirely within the endoplasmic reticulum, while Fbxo7 is present throughout the cell, shuttling between the nucleus and cytoplasm in a cell cycle-dependent fashion [71,73,107]. At present, it is unclear what proportions of Fbxo7 and PI31 exist in heterodimeric complexes or if this changes in different cell types or culture conditions.

The clearest evidence for a relationship between PI31 and a partial Fbxo7 orthologue, Nutcracker, in the regulation of proteasomes comes from studies in *Drosophila* [52]. The effects of their loss are seen during ‘individualization’, the final stage of spermatogenesis, which is a process that uses components of the apoptotic system to purge organelles and excess cytoplasmic volume to produce mature sperm [108,109]. Bader *et al*. [52] found that DmPI31 stability was regulated by Nutcracker, and like their mammalian counterparts, the two proteins interact via their FP domains. This interaction inhibited DmPI31 cleavage, promoting proteasome activity and caspase activation. Intriguingly, the Nutcracker F-box domain was essential for DmPI31 stabilization, even though it did not mediated their interaction and DmPI31 was not found to be a substrate for Nutcracker-dependent ubiquitination.

Nutcracker may also have an additional role to play in maintaining the delicate balance of caspase activity that is required during individualization. In a further parallel with mammalian Fbxo7 biology, Nutcracker also interacts with dBruce, a known regulator of spermatogenesis and a member of the IAP family of apoptosis inhibitors to which the Fbxo7 substrate cIAP1 belongs [108]. dBruce is a giant protein (approx. 500 kDa), with two principal anti-apoptotic activities: its N-terminal BIR domain enables it to function as a caspase inhibitor and its C-terminal UBC domain provides E2 ubiquitin conjugation activity, which has been shown to promote ubiquitination of the pro-apoptotic factor, Reaper [109,110]. Currently, the functional consequences of the Nutcracker : dBruce interaction are not clear. dBruce may indeed be a substrate of SCF^Nutcracker^, but on the other hand it may simply act as an E2 enzyme for the complex in this setting, interacting directly with Nutcracker during ubiquitin transfer. Alternatively, Nutcracker could instead use its interactions with DmPI31 and dBruce, positive and negative regulators of caspases, respectively, to fine-tune their activity during spermatogenesis.

Most recently, Cho-Park & Steller [111] have shown that DmPI31 also controls the constitutive proteasome by regulating the attachment of the 19S regulatory particle to the 20S core. They showed that PI31 binding activity for the proteasome is ‘switchable’ and is controlled by ADP-ribosylation, promoting assembly of active 26S proteasomes. It has been postulated that this mechanism might tie proteasome activity to cellular metabolism as NAD^+^ is used for ADP-ribosylation of PI31 [111,112]. As a strong binding partner for DmPI31, it is possible that Nutcracker might impact on this aspect of PI31 regulation.

In *Drosophila*, dramatic phenotypes for *DmPI31* and *nutcracker* loss were reported only in the testes. The requirement for PI31 and Fbxo7 in mammalian systems may also be tissue specific, necessitated in cells that stringently require high levels of proteasome activity. As mutations in *FBXO7* have now been linked with PD, a disease in which UPS dysfunction and protein aggregation are potential contributors to its aetiology, it is tempting to speculate that Fbxo7 regulation of proteasome activity might also play a part in this disease. It is possible that in PD, as misfolded proteins accumulate and UPS stress mounts, the demand to ramp up proteasomal activity cannot be met by cells that have substandard Fbxo7 activity. This would hint at an underlying lack of fitness in the UPS. An alternative hypothesis is that the dimerization of PI31 with Fbxo7 might prevent or alter its SCF-dependent functions or its other atypical roles; thus PI31 might act as an inhibitor or refiner of Fbxo7-dependent substrate ubiquitination or its cell cycle regulatory roles.

## 9. Fbxo7 mutations cause Parkinson's disease

The identification of mutations within *FBXO7* in patients presenting with an early-onset form of PD opened up new questions about the role of Fbxo7 in the preservation of neuronal health [75,113,114]. Whole-genome SNP arrays were instrumental in the discovery of the first disease-associated variant of the *FBXO7* gene, revealing a homozygous mutation (R378G) in an Iranian family [114]. This was quickly followed by the identification of other mutations, including a homozygous truncating mutation (R498X) in an Italian family and compound heterozygous mutations consisting of a splice-site (IVS7 + 1G/T) and point mutation (T22M) in a Dutch family [75]. Another heterozygous mutation (R481C) was reported in an Italian family [115]. However, affected family members were also found to have a homozygous mutation within *PARK9* (G877R), so it is unclear whether the mutation of a single *FBXO7* allele contributed to the disease phenotype. What is striking about the mutations discovered to date is that they are distributed across the many functional domains of the protein (figure 3*c*), suggesting that a number of Fbxo7 functions are relevant to PD. Indeed, early characterization of the R378G mutant has shown that this mutation reduces Fbxo7 affinity for Skp1 [107]. In addition, as the R498X mutation removes 24 amino acids from the substrate-binding domain, these mutants hint that loss of Fbxo7's SCF-dependent E3 ligase activity will be an important aspect in the pathogenesis of the disease.

As a bona fide PD-associated gene, *FBXO7* has been designated ‘*PARK15*’ and joins a small family of ‘PARK’ genes. These genes all have confirmed genetic association with PD and are classified as autosomal dominant or recessive. Dominant mutation of a single copy of *PARK1*, which encodes α-synuclein, can produce a gain-of-function, whereby the protein becomes prone to aggregation. α-synuclein is the main constituent of Lewy bodies, the intracellular proteinaceous plaques observed in the brains of idiopathic PD patients at autopsy. Lewy bodies are thought to develop from aggregated, ubiquitinated proteins, which accumulate at the microtubule-organizing centre (MTOC) of the cell in structures known as aggresomes [116,117]. Whether these deposits are part of a pathological process or a protective means to sequester cytotoxic oligomers of misfolded proteins remains controversial. In any case, they represent a residual signature for the inefficient functioning of the proteasome and/or autophagy pathways in the neurons of patients [118–120]. One study has been published suggesting that Fbxo7 may be involved in Lewy body formation [121]. Using immunohistochemistry, its expression was detected throughout ‘normal’ brain tissue, but was surprisingly more abundant in the neocortex, putamen and cerebellum than in the substantia nigra. Fbxo7 was also found in α-synuclein-positive Lewy bodies in idiopathic brain tissue [121]. This study lacked experiments that addressed the mechanism of Fbxo7 deposition in intracellular aggregates, so it is not known whether Fbxo7 is actively involved in the aggregation and disposal of misfolded proteins or whether Fbxo7 is merely a passenger that is itself prone to aggregation in stressed neurons. Answers to these questions await future molecular studies and analysis of PARK15 patient samples.

## 10. Fbxo7 as a regulator of mitophagy

*FBXO7* is mutated in an autosomal recessive fashion, like several other PARK genes including *Parkin* (*PARK2*) and *PTEN-induced kinase 1 (PINK1; PARK6*), so it is thought that their normal functions are reduced or lost as a result of mutation. Parkin is a single-subunit RING-type E3 ubiquitin ligase that contributes to neuronal health by initiating the bulk-disposal of misfolded or aggregated proteins, and by regulating the mitochondrial quality control process, known as ‘mitophagy’ [122–125]. Mitophagy is a form of selective macroautophagy that enables the cell to constantly survey its mitochondrial network, identifying and removing damaged, depolarized mitochondria. This process runs continually, working alongside mitochondrial biogenesis to meet the energy requirements of the cell while minimizing its oxidative burden. Maintaining this balance is likely to be of particular importance for the long-lived dopaminergic neurons of the substantia nigra, which are lost during the progression of PD. These highly specialized neurons have massive, unmyelinated axonal arborizations that place extraordinary demands on their mitochondria [126,127]. The involvement of Parkin and PINK1 in the mitophagy pathway was uncovered in a series of genetic studies in *Drosophila* showing that loss of either gene resulted in identical mitochondrial defects in flight muscles and sperm. Their epistatic relationship was discovered when over-expression of Parkin was shown to rescue *PINK1* null flies but not *vice versa* [128]. PINK1 acts as the sensor of mitochondrial membrane potential (*Δψ*m), and under healthy conditions it is a highly labile protein, constitutively cleaved and shed from mitochondria before being degraded in the cytosol. However, when *Δψ*m is lost, PINK1 accumulates and is integrated into the outer mitochondrial membrane. PINK1 recruits and activates cytosolic Parkin by phosphorylation [129,130], which directly or indirectly stimulates the Lys48 and Lys63 polyubiquitination of a number of proteins. This leads to the degradation of mitofusin (Mfn) 1/2, Drp1 and voltage-dependent anion channel 1 (VDAC1) [131–134], initiating the fragmentation and isolation of depolarized regions of the mitochondrial network. The coating of Lys63 polyubiquitin enables the LC3-adaptor protein, p62/SQSTM1, to bind these mitochondrial fragments, and traffic them to the nuclear periphery where they form aggresome-like structures and are eventually engulfed into autophagosomes.

Burchell *et al.* [77] have shown that Fbxo7 is also a component of this pathway (figure 3*d*), with Fbxo7 interacting directly with Parkin via its Ubl domain, helping to recruit it to mitochondria to initiate mitophagy. The T22M mutation in the Ubl of Fbxo7 ablates its interaction with Parkin, and thus prevents its recruitment to depolarized mitochondria. Fbxo7 was also required for the efficient ubiquitination of Mfn1 and the recruitment of p62. Importantly, the expression of human Fbxo7 rescues the phenotypes of *parkin* loss in a *Drosophila* model of neurodegeneration [77]. These data strongly suggest that the Parkin–Fbxo7 interaction is important for neuronal health.

In addition to interacting with Parkin, Fbxo7 also directly interacts with PINK1, via a domain that encompasses and extends beyond the Parkin binding site in the Fbxo7 Ubl domain (figure 3*c*). *In vitro* binding studies showed neither a cooperative nor a competitive interaction among the three proteins, suggesting that Fbxo7 may act as a scaffold to facilitate PINK1-mediated phosphorylation and activation of Parkin. Parkin is clearly downstream of PINK1 loss in the *Drosophila* model. However, the expression of human Fbxo7 did not rescue the mitochondrial defects of *PINK1* null flies. Fbxo7 is not required for PINK1 accumulation at depolarized mitochondria, but these results might be explained by the requirement for PINK1 activity for rescue by Fbxo7 to take place.

At present, it is not known whether Fbxo7 solely acts as a recruitment factor for Parkin during mitophagy or if it has a more elaborate role to play. However, as Fbxo7 can compensate for all of the mitochondrial defects resulting from *parkin* loss in *Drosophila*, this would indicate that all the critical ubiquitination targets/functions for Parkin are fulfilled in the fly model. Do Fbxo7 and Parkin have overlapping substrate profiles in mammalian cells or does Fbxo7 have its own PD-relevant substrates? Certainly, the other pathogenic mutations within Fbxo7 suggest that its E3 activity is important for neuronal health even in the presence of Parkin, so discovering their identity is of great interest. Whichever substrate(s) are discovered to be the key targets, the finding that three PARK genes are involved in a common function to regulate mitochondrial homeostasis firmly focuses the PD field on the pathways regulating mitochondrial health for investigating the possibilities of targeted therapeutics and diagnostics.

## 11. Concluding remarks

With such a multifunctional protein that is relevant to human disease, there are a myriad of questions regarding the function, regulation and specificity of Fbxo7. For example, the sheer diversity of Fbxo7's canonical and non-canonical interactions raises questions of how Fbxo7 activity is dictated at a given moment. In other words, does Fbxo7 participate in all of these functions at all times, or do some activities pre-dominate over others? We note that many of Fbxo7's functions are restricted, spatially or temporally, at particular organelles, or in signalling hubs. For example, the assembly of active Cdk6 with D-type cyclins happens in a short window during G1 phase, and the need for boosting the proteasome activity may be a transient response to pathogens or other cellular stresses. In addition, although mitophagy in the Burchell *et al*. study used chemicals to induce the mass depolarization of the mitochondria, this is unlikely to occur so profoundly in cells. Instead, mitophagy more probably occurs at a low level and as a last resort to purge the network of irreparably damaged mitochondria. We speculate that Fbxo7 participates in mitophagy in other tissues as well as neurons, and it is also possible that in neurons, the atypical functions of Fbxo7, like proteasome regulation may also come into play. For example, where poor Fbxo7 function leads to mitochondrial dysfunction, this may contribute to the production of reactive oxygen species, which causes protein damage, misfolding and aggregation, and eventually overburdening of the UPS. Suboptimal Fbxo7 may contribute to the poor coping of proteasomes to this increased demand, directly or through its interactions with PI31. Additionally, Fbxo7's other atypical roles may also conceivably contribute to the development of PD, like in cell cycle regulation, where inappropriate cell cycle entry has been linked to the death of neurons, or in the NF-κB pathway, which has been linked to inflammation in the brain. Perhaps, it is due to the multifunctional character of *PARK* genes, like Fbxo7 and Parkin, that when they are defective, they disable numerous pathways, causing disease.

Fbxo7 is certainly an extraordinary case for the cell getting many disparate functions from a single protein and possibly for linking cellular pathways to each other. If the other 68 FBPs in the cell are so intricately engineered, there will be much to functionally dissect for the future. The challenge will be to understand the tremendous complexity and interplay of all the different types of activities of FBPs in the context of both development and disease, and tease out which aspects of their functions we can influence to benefit patients.
